# Is There a Link Between TSH Levels and Schizophrenia? A Systematic Review and Meta-Analysis

**DOI:** 10.3390/jcm14175959

**Published:** 2025-08-23

**Authors:** Elisa Gatta, Francesco Dondi, Ilenia Pirola, Andrea Delbarba, Virginia Maltese, Pietro Bellini, Massimiliano Ugoccioni, Irene Silvestrini, Mario Rotondi, Francesco Bertagna, Carlo Cappelli

**Affiliations:** 1 Department of Clinical and Experimental Sciences, SSD Endocrinologia, University of Brescia, ASST Spedali Civili, 25121 Brescia, Italy; elisa.gatta@unibs.it (E.G.); virginia.maltese@asst-spedalicivili.it (V.M.); 2 Centro per la Diagnosi e Cura delle Neoplasie Endocrine e delle Malattie della Tiroide, University of Brescia, 25121 Brescia, Italy; 3 Nuclear Medicine, University of Brescia, ASST Spedali Civili, 25121 Brescia, Italypietro.bellini@asst-spedalicivili.it (P.B.); 4 Department of Internal Medicine and Therapeutics, University of Pavia, 27100 Pavia, Italy; 5 Laboratory for Endocrine Disruptors, Unit of Internal Medicine and Endocrinology, Istituti Clinici Scientifici Maugeri IRCCS, 27100 Pavia, Italy

**Keywords:** thyroid, thyroid hormones, schizophrenia

## Abstract

**Background**: Thyroid dysfunction and psychiatric disorders often coexist, raising interest in their potential interplay. In particular, the relationship between thyroid-stimulating hormone (TSH) levels and schizophrenia has been investigated, though findings remain inconsistent. We performed a systematic review and meta-analysis to clarify whether TSH levels differ in patients with schizophrenia compared with healthy controls. **Methods**: A systematic search of PubMed/MEDLINE, Scopus, and Web of Science was conducted up to May 2025. Eligible studies were selected based on predefined criteria according to the PICO framework: What are the TSH levels in first-episode, drug-naïve patients diagnosed with schizophrenia compared with healthy subjects, and do TSH levels influence different psychiatric phenotypes? PRISMA guidelines were followed. Study quality and risk of bias were assessed using QUADAS-2. **Results**: Of 2068 records screened, 35 studies met the inclusion criteria. Quality assessment revealed a generally unclear risk of bias, with few studies showing a high risk. The meta-analysis included 3669 patients and 1391 controls from ten Asian, eight European, and three North American studies. TSH levels were similar between patients and controls (SMD = –0.059 mIU/L; 95% CI: –0.260 to 0.141), with substantial heterogeneity (I^2^ = 84%, *p* < 0.001). **Conclusions**: This meta-analysis found no significant association between TSH levels and schizophrenia, despite decades of research and methodological diversity.

## 1. Introduction

Thyroid dysfunction refers to temporary or chronic conditions that result in decreased or increased production of thyroid hormones (THs). THs play a critical role in human homeostasis, impacting a wide array of tissues including the heart, muscle, bone, and brain. In particular, they are crucial for the development of the mammalian brain, acting on the migration and differentiation of neural cells, synaptogenesis, and myelination [1]. Moreover, tetraiodothyronine (T4) and triiodothyronine (T3) are related to short- and long-term brain changes, including neuronal plasticity processes, angiogenesis, and neurogenesis in adults [2]. T4 and T3 regulate thyroid-stimulating hormone (TSH) secretion through a negative feedback mechanism. Due to the sensitivity of TSH secretion to small fluctuations in free T4 levels, abnormal TSH concentrations are often detected before changes in free T4 in both hypothyroidism and hyperthyroidism. There is a log–linear relationship between T3/T4 and TSH, meaning even minor variations in T3/T4 can lead to significant changes in TSH levels [3]. This supports the role of TSH, in the absence of hypothalamic/pituitary disease, as the most sensitive marker of thyroid function [3,4].

Schizophrenia (SHZ) is characterized by substantial impairments in reality testing and changes in behavior, which appear in both positive symptoms (such as persistent delusions, continual hallucinations, disorganized thinking, grossly disorganized behavior, and experiences of passivity and control) and negative symptoms (such as blunted or flat affect, avolition, and psychomotor disturbances). These symptoms present with enough frequency and intensity to deviate from expected cultural or subcultural norms [5]. It is one of the most disabling and economically devastating medical conditions, ranked by the World Health Organization among the top 10 diseases contributing to the global burden of disease [6].

Emerging evidence suggests that THs may influence neurodevelopmental pathways, neurotransmitter systems (such as dopaminergic and serotonergic circuits), and neuroinflammatory processes implicated in SHZ [7]. Alterations in thyroid function, even within the reference range, have been associated with changes in mood, cognition, and psychomotor activity, which could overlap with or exacerbate SHZ symptoms [7]. Notably, a study conducted ten years ago reported a relationship between THs and suicide risk in patients with SHZ [8].

Given the high prevalence of thyroid dysfunction and psychiatric disorders, it is quite common for individuals to be affected by both conditions simultaneously. In fact, it has been reported that SHZ patients have hypothyroidism and hyperthyroidism in 25% and 4% of cases, respectively [9]. On the other hand, it is known that antipsychotic medications, particularly second-generation agents (i.e., olanzapine, aripiprazole), have been associated with alterations in thyroid function, most commonly subclinical hypothyroidism [10,11].

Many studies have examined the role of TSH levels in patients with psychiatric disorders, with conflicting results [7,12,13]. Therefore, we conducted this systematic review and meta-analysis to explore the association between TSH levels and schizophrenia. The aim was to compare TSH levels in first-episode, drug-naïve patients diagnosed with SHZ to those of healthy controls, and to investigate whether TSH levels are associated with different psychiatric phenotypes and/or disease severity in SHZ.

## 2. Materials and Methods

### 2.1. Search Strategy and Inclusion Criteria

A wide literature search of the PubMed/MEDLINE, Scopus, and Web of Science databases was conducted, based on the Preferred Reporting Items for Systematic Reviews and Meta-Analyses (PRISMA) methodological guidelines.

The review questions were defined based on the “Population, Intervention, Comparator, Outcome” framework (PICO): What are the TSH levels in first-episode drug-naïve (FEDN) patients diagnosed with schizophrenia (SHZ) (patients/population) compared with healthy subjects (comparator), and do TSH levels influence different psychiatric phenotypes (outcome)?

The algorithm used for the research was the following: (“thyroid”) AND (“schizophrenia”).

The search was updated until 31 May 2025. Only articles in English were considered, and preclinical studies, conference proceedings, reviews, or editorials were excluded. To expand our search, the references of the retrieved articles were also screened for additional papers.

### 2.2. Eligibility Criteria

The eligibility criteria were chosen by taking into account the review question. Clinical studies reporting TSH levels in patients diagnosed with SHZ were deemed eligible for inclusion in this systematic review. Exclusion criteria for the systematic review (qualitative analysis) were reviews, letters, comments, and editorials on the topic of interest, case reports, or small case series (fewer than five enrolled patients) on the analyzed topic (as these articles are characterized by poor-quality evidence and are typically affected by publication bias), as well as original articles dealing with different fields of interest. Moreover, an exclusion criterion was the presence of non-thyroidal illness syndrome (NTIS), a condition characterized by altered thyroid hormone levels with unchanged TSH levels. In agreement, only studies reporting the results of the thyrotropin-releasing hormone (TRH) test and/or the levels of TSH and thyroid hormone, including free T4 (FT4) and/or T4 and/or free T3 (FT3) and/or T3 in serum or plasma samples were included in the quantitative analysis. In addition, no studies including patients with known thyroid diseases were included in the quantitative analysis.

### 2.3. Study Selection

E.G. and V.M. independently read the titles and abstracts of the records generated by the search algorithm. They then determined which studies were eligible based on predefined criteria.

### 2.4. Reporting and Quality Assessment

The protocol of this systematic review is registered in PROSPERO (CRD420251077986) and follows the PRISMA (Preferred Reporting Items for Systematic Reviews and Meta-Analyses) guidelines. The quality assessment of the studies, including the risk of bias and applicability concerns, was carried out using the Quality Assessment of Diagnostic Accuracy Studies version 2 (QUADAS-2) evaluation [14].

### 2.5. Data Extraction

Two reviewers (E.G. and V.M.) independently extracted data from each included study; any discrepancies in data extraction were resolved by discussion. The data were collected from all of the included studies, taking advantage of full-text, tables, and supplemental material concerning general study information (authors, publication year, country, study design, funding sources), patients’ characteristics (sample size, age, clinical setting, diagnosis, therapies), and TSH levels. The main findings of the articles included in this review are reported in the Results Section.

### 2.6. Statistical Analysis

The data from the included studies were utilized, considering each study’s relative importance, by employing a random-effects statistical model due to the high heterogeneity in the analyzed studies. Furthermore, the study included the provision of 95% confidence interval values, which were subsequently visually represented through forest plots. The I-square (I^2^) index, also known as the inconsistency index, was employed to assess the level of statistical heterogeneity within the papers included in the analysis. Statistical heterogeneity was considered significant if the I-square index exceeded 50%. The software OpenMeta [Analyst]^®^ (version 3.13), supported by the Agency for Healthcare Research and Quality (AHRQ) in Rockville, MD, USA, was utilized to calculate the pooled values of standardized mean differences (SMD).

## 3. Results

### 3.1. Literature Search

**Figure 1:**
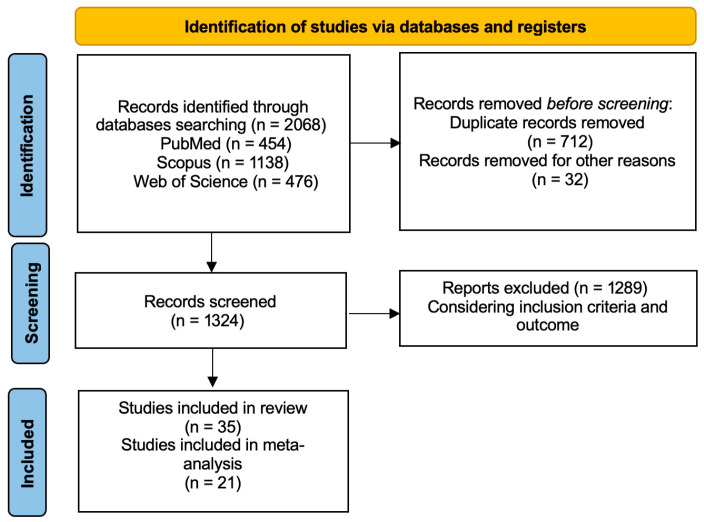
Flowchart of the search for eligible studies on the impact of thyroid function on psychiatric diseases.

A total of 2068 articles were identified through the computer literature search. By reviewing the titles and abstracts, 1289 articles were excluded because the reported data were not within the field of interest for this review. Consequently, 35 articles were selected and evaluated in the review [8,15,16,17,18,19,20,21,22,23,24,25,26,27,28,29,30,31,32,33,34,35,36,37,38,39,40,41,42,43,44,45,46,47,48] (Figure 1).

**Figure 2:**
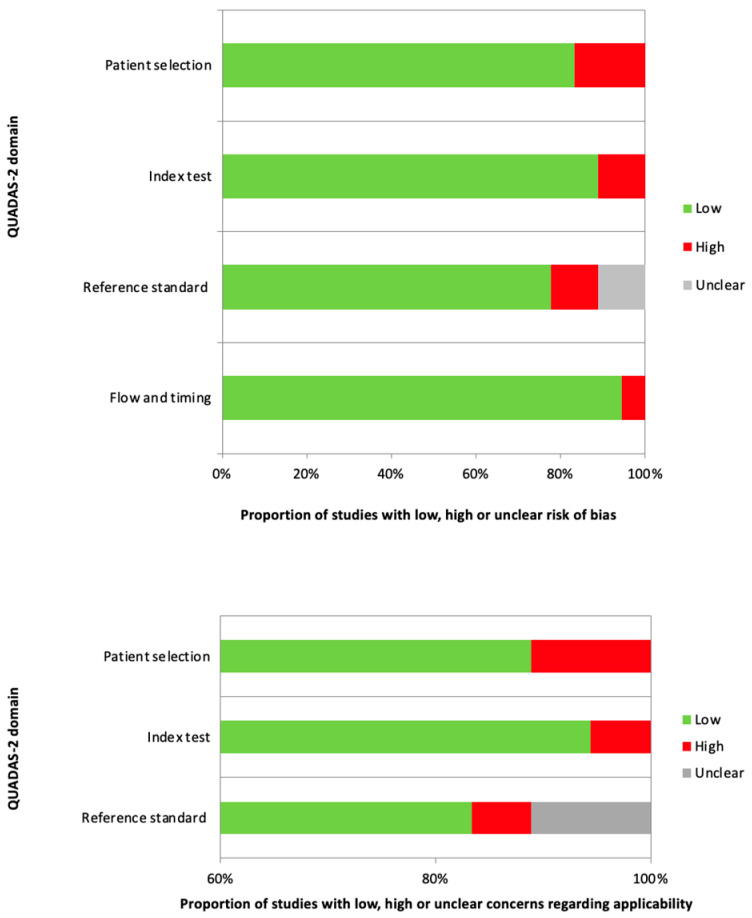
QUADAS-2 quality assessment of risk of bias and applicability concerns in the studies considered in the review.

In general, the quality assessment using the QUADAS-2 evaluation underlined the presence of unclear risk of bias and applicability concerns in some of the studies concerning patient selection, index test, reference standard, and flow and timing. Nevertheless, only a small number of studies were characterized by the presence of a high risk of bias or applicability (Figure 2).

The main characteristics of the studies and their results are briefly presented in Table 1 and Table 2.

In detail, 18 studies were of a retrospective nature, 11 had a prospective design, and six were interventional studies.

### 3.2. Qualitative Analysis

The available data on 9163 (46% male) patients affected by SHZ were retrieved (Table 1 and Table 2).

Forty years ago, Banki et al. investigated the TRH–TSH response in SHZ patients. The authors showed no differences in ΔTSH between 24 SHZ women and 15 healthy controls (ΔTSH 7.97 ± 5.5 mIU/L vs. 14.71 ± 8.33 mIU/L, respectively, *p* ≤ 0.001) [11]. These data were confirmed in other interventional studies in the years to come [16,17,35].

The difference in serum TSH levels between naïve SHZ patients compared to healthy subjects has been extensively evaluated, with discordant results [8,15,16,17,18,19,20,22,24,28,29,30,34,35,38,40,41,45,46,49,50]. The latest study by Glodek et al. confirmed a long series of previous ones [15,17,19,20,22,24,25,26,27,28,30,35,38,40,45,46], showing no TSH differences between SHZ patients and healthy controls (1.5 ± 0.78 mIU/L vs. 1.98 ± 0.54 mIU/L, respectively, *p* = 0.572) [45]. Conversely, Bicikova et al., investigating a few biochemical markers in drug-naïve SHZ patients, showed lower TSH levels than healthy controls [median: 1.21 mIU/L (IQR 0.80–1.80) vs. 1.84 mIU/L (IQR 1.4–2.36), respectively, *p* < 0.05] [21]. The data were supported by several studies in subsequent years [18,23,29,32,36]. In addition, very recently, Esposito et al. showed in a large multicenter retrospective study a significant difference in TSH levels according to gender in SZH patients. Specifically, the mean TSH levels in 233 female patients were significantly higher than those in 322 male patients (TSH 2.89 ± 3.56 mIU/L vs. 1.90 ± 1.24 mIU/L, respectively, *p* = 0.008) [43].

Very recently, Jiang et al. showed a strong relationship between TSH levels and disease severity (mild 2.08 ± 0.52 vs. moderate 2.88 ± 0.85 vs. severe 3.41 ± 0.99, *p* < 0.001) [48]. Conversely, Jose et al. did not show any correlation between TSH levels and SHZ symptoms (*r* = −0.057 and *r* = 0.098, positive and negative, respectively, *p* = NS). However, the authors showed lower TSH levels in patients attempting suicide than in those not attempting (1.52 ± 0.63 mIU/L vs. 1.89 ± 1.76 mIU/L), without reaching statistical significance [8].

### 3.3. Quantitative Analysis

**Figure 3:**
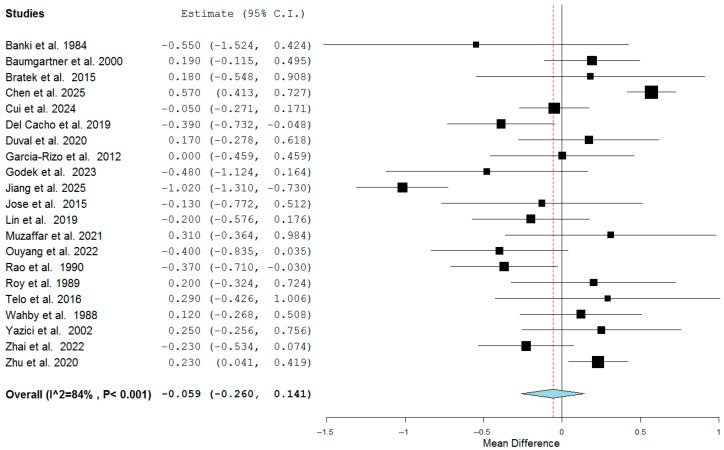
Forest plot of the meta-analysis of serum concentrations of thyroid-stimulating hormone (TSH) in patients with first-episode schizophrenia compared with healthy controls [8,15,16,17,18,19,20,22,24,28,29,30,34,35,38,40,41,45,46,47,48]. Each black square represents the effect estimate of a single study, with the size of the square proportional to its statistical weight. Horizontal lines indicate 95% confidence intervals. The red vertical dashed line represents the line of no effect (mean difference = 0). The blue diamond at the bottom illustrates the pooled overall effect estimate, with its width corresponding to the 95% confidence interval.

The available data retrieved from 21 studies [8,15,16,17,18,19,20,22,24,28,29,30,34,35,38,40,41,45,46,47,48] comparing TSH levels between SHZ patients and healthy subjects were pooled using a random-effects model. A total of 3669 patients and 1391 healthy controls from ten studies from Asia, eight from Europe, and three from North America were included. Five studies were interventional in nature, seven retrospective, and nine prospective. The meta-analysis showed that FEDN SHZ patients displayed superimposable serum TSH levels compared to healthy subjects (SMD = −0.059 mIU/L, C.I. 95%:−0.260 to 0.141) with significant heterogeneity across studies (I^2^ = 84%, *p* < 0.001) (Figure 3).

## 4. Discussion

The present systematic review, and particularly our meta-analysis, did not show any association between TSH levels and FEDN SHZ patients.

Thyroid hormones are fundamental to brain development and function, regulating key neurobiological processes such as synaptogenesis and myelination—both critical for learning and memory [1,51]. Consequently, both hypo- and hyperthyroid states can adversely affect neurocognitive performance. For instance, maternal thyroid hormone insufficiency during pregnancy has been associated with cognitive impairments, intellectual disabilities, language delays, and attentional deficits in offspring [52,53]. Similarly, hyperthyroidism has been linked to reduced cognitive abilities [54], and even subclinical hyperthyroidism has been implicated in a progressive decline in cognitive function [55].

Clinically relevant hyperthyroidism might manifest with psychotic symptoms [56,57,58,59,60,61], although studies investigating TH in persons with psychotic disorders have provided mixed findings [62]. We must also underline that thyroid disorders and schizophrenia may share overlapping positive and negative symptoms—such as psychomotor slowing, apathy, cognitive impairment, and mood disturbances—potentially complicating both diagnosis and treatment [63]. This overlap underlines the necessity of an accurate diagnostic process. However, TH are known to modulate serotonergic and γ-aminobutyric acid (GABA) pathways, which can indirectly influence the clinical symptoms of schizophrenia [63]. Additionally, TH receptors are localized to limbic structures implicated in mood regulation [64], and thyronine can specifically bind to other defined neurotransmitter receptors, including those in GABAergic, catecholaminergic, glutamatergic, and cholinergic systems [65].

Schizophrenia (SHZ) is among the most disabling and economically catastrophic medical disorders. It is ranked by the World Health Organization as one of the top 10 illnesses contributing to the global burden of disease [6]. Additionally, it appears to be a uniquely human condition, limiting the utility of animal models and complicating the elucidation of its etiopathophysiology [66]. However, THs seem to profoundly shape brain development and function through multiple molecular mechanisms that are highly relevant to SHZ pathophysiology. They act via nuclear receptors (TRα/TRβ) to regulate gene expression involved in neuronal differentiation, synaptogenesis, and myelination [1,63]. Local control of active T3 in the brain by deiodinase enzymes—particularly DIO2—ensures region-specific thyroid signaling, a process that may be disrupted in SHZ [67]. In addition, THs also modulate key neurotransmitter systems—dopaminergic, serotonergic, glutamatergic, and GABAergic—which are central to SHZ’s neurobiology [1,63,68].

The interaction between thyroid function and the manifestation of positive and negative symptoms in schizophrenia has also been explored, with studies suggesting a potential role for thyroid hormones in behavioral dysregulation related to the brain’s reward system [69].

In addition, many studies have investigated the relationship between SHZ and TSH levels with discordant results [8,15,16,17,18,19,20,21,22,23,24,25,26,27,28,29,30,31,32,33,34,35,36,37,38,39,40,41,42,43,44,45,46,47,48]. For example, Chen et al., very recently, showed in a large cross-sectional study that people experiencing a first episode of schizophrenia exhibit lower TSH values than healthy controls [47]. In contrast, Melamed et al. showed an increased incidence of hypothyroidism in patients with schizophrenia [13]. Moreover, recently, a population-based study showed an increased rate of hypothyroidism in patients with schizophrenia after, but not before, the onset of the disease [70]. On the other hand, recently, Freuer and Meisinger, in a very elegant bidirectional two-sample Mendelian randomization study, showed that genetic liability for hypothyroidism was inversely associated with schizophrenia, with an inverse relationship between hypothyroidism and schizophrenia [7].

Our meta-analysis, taking into account 3669 patients and 1391 healthy controls from 21 studies obtained from Asia, North America, and Europe, clearly showed comparable TSH levels among patients diagnosed with SHZ and healthy subjects at the onset of disease (Figure 3). In other words, these data suggest no correlation between TSH levels and schizophrenia, at least at the onset of disease. Nevertheless, it has been shown that TSH levels could be correlated with disease severity [48], and perhaps inversely correlated with suicide risk [8]. If this finding is confirmed, we can hypothesize the effect of drugs (i.e., thioamides and/or thyroxine) on TSH levels in euthyroid psychiatric patients. However, our data do not show any correlation between TSH levels and either the severity of schizophrenia or the increased risk of suicide.

## 5. Conclusions

We have reviewed the literature from the past forty years regarding the possible interplay between TSH and schizophrenia. Amidst the flashes and thunder, our data do not show any correlation between TSH levels and schizophrenia and therefore do not support routine TSH screening in these patients.

## Figures and Tables

**Table 1:** Characteristics of the human studies included in the review.

First Author	Ref. N.	Year	Country	Study Design	N. Pts.	Age	Sex M:F
Banki	[15]	1984	Hungary	Interventional	24	41.0 ± 13.0	0/24
Wahby	[16]	1988	United States	Interventional	37	35.5 ± 3.9	37/0
Roy	[17]	1989	United States	Interventional	14	25.4 ± 4.4	7/7
Rao	[18]	1990	German	Retrospective cross-sectional	110	34 ± 13	58/52
Baumgartner	[19]	2000	Germany	Prospective cross-sectional	31	29.2 ± 9.1	22/9
Yazici	[20]	2002	Turkey	Interventional	58	32.5 ± 11.4	35/23
Bicikova	[21]	2011	Czech Republic	Interventional	22	32.6 ± 7.4	13/9
Garcia-Rizo	[22]	2012	Spain	Prospective cross-sectional	33	28.6 ± 7.1	20/13
Wysokiński	[23]	2014	Poland	Retrospective cross-sectional	769	40.0 ± 16.2	381/388
Bratek	[24]	2015	Poland	Prospective cross-sectional	15	36.6 ± 7.5	15/0
Degner	[25]	2015	Germany	Prospective cross-sectional	19	43.5	7/12
Jose	[8]	2015	India	Retrospective cross-sectional	38	26.8 ± 4.4	38/0
Liang	[26]	2016	China	Retrospective observational	219	61.1 ± 6.6	0/219
Petrikis	[27]	2016	Greece	Retrospective observational	40	32.5 ± 9.8	27/13
Telo	[28]	2016	Turkey	Retrospective cross-sectional	63	44.7 ± 10.4	31/32
Del Cacho	[29]	2019	Spain	Prospective cross-sectional	61	24.6 ± 9.3	38/23
Lin	[30]	2019	Taiwan	Prospective cross-sectional	69	41.8 ± 10.4	40/29
Kalinowska	[31]	2019	Poland	Retrospective observational	106	41.89 ± 9.7	42/64
Kornetova	[32]	2020	Russia	Retrospective cross-sectional	156	NA	68/88
Petruzzelli	[33]	2020	Italy	Retrospective observational	30	15.4 ± 1.7	6/16
Zhu	[34]	2020	China	Retrospective cross-sectional	486	39.3 ± 12.6	292/194
Duval	[35]	2021	France	Interventional	13	31.1 ± 10.3	13/0
Li	[36]	2021	China	Retrospective cross-sectional	83	34 (IQR 29–47)	37/46
Makarow-Gronert	[37]	2021	Poland	Retrospective cross-sectional	59	NA	23/36
Muzaffar	[38]	2021	United States	Prospective cross-sectional	19	NA	19/0
Zhao	[39]	2021	China	Retrospective observational	2022	31.3 ± 10.8	801/1221
Ouyang	[40]	2022	China	Prospective observational	46	25.33 ± 5.75	22/24
Zhai	[41]	2022	China	Retrospective observational	235	26.5 ± 9.5	87/148
Zhao	[42]	2022	China	Retrospective observational	1302	32.5 ± 11.1	455/847
Esposito	[43]	2023	Italy	Retrospective observational	555	43.4 ± 13.9	322/233
Li	[44]	2023	China	Retrospective observational	89	21.83 ± 7.94	50/39
Głodek	[45]	2023	Poland	Prospective cross-sectional	47	39.1 ± 11.4	25/22
Cui	[46]	2024	China	Prospective cross-sectional	1186	29.27 ± 9.35	536/650
Chen	[47]	2025	China	Prospective cross-sectional	1007	47.01 ± 13.02	602/405
Jiang	[48]	2025	China	Retrospective observational	100	34.6 ± 10.4	46/54

Ref.: references; N.: number; Pts.: patients; M: male; F: female; NA: not available.

**Table 2:** Summary of studies assessing TSH levels in first-episode drug-naïve patients with schizophrenia.

First Author	Patients’ Characteristics	TSH Levels	Main Findings
Banki [15]	Hospitalized adult women	1.89 ± 1.37	No difference in TRH–TSH response was found between SHZ and controls.
Baumgartner [19]	Hospitalized adult patients for acute SHZ	1.2 ± 0.7	No difference in TSH was found among SHZ patients and healthy controls.
Bicikova [21]	Patients diagnosed with SHZ	1.21 (IQR 0.8–1.8)	SHZ patients showed lower TSH levels and higher AbTPO titers.
Bratek [24]	Hospitalized adult patients	1.76 ± 1.08	No difference in TSH was found among SHZ patients and healthy controls.
Chen [47]	Hospitalized adult patients	2.05 ± 2.25	Sex differences exist in thyroid hormone T3 levels in people with schizophrenia.
Cui [46]	Aged below 50 adult patients	1.89 ± 1.74	No differences in TSH levels were found among SHZ and healthy controls.
Degner [25]	Adult outpatients without previously diagnosed thyroidal diseases.	1.6 ± 1.5	No differences in TSH levels were found among BD, MDD, and SHZ patients; AbTPO levels were higher in MDD and BD compared with SHZ.
Del Cacho [29]	Inpatients hospitalized for acute illness	1.47 ± 0.95	Significantly lower TSH levels were found in acute SHZ patients.
Duval [35]	Adult male hospitalized patients who underwent TRH–TSH stimulation	1.30 ± 0.69	TSH response is unaltered in schizophrenia patients.
Esposito [43]	Adult inpatients diagnosed with SHZ	2.33 ± 2.56	TSH levels were significantly higher, and hypothyroidism was more frequent, in women inpatients than men.
Garcia-Rizo [22]	SHZ outpatients	1.8 ± 1.0	No differences in TSH levels were found among SHZ patients and healthy controls.
Głodek [45]	Adults hospitalised in the Department of Adult Psychiatry with age between 18 and 65	1.50 ± 0.78	No significant differences in thyroid function were found between BD and schizophrenia patients.
Jiang [48]	Adult inpatients diagnosed with SHZ	3.10 ± 0.90	The serum levels of T3, FT3, FT4, TSH, and cortisol in the schizophrenia group were lower than those in the control group (*p* < 0.05).
Jose [8]	SHZ patients aged 18 to 45 years	1.82 ± 1.61	fT4 increased in patients with schizophrenia as compared with controls and in those with suicidal ideation.
Kalinowska [31]	Outpatients aged 18 to 70 years during the remitted state of the disease	2.28 ± 1.46	An association between TSH values and metabolic syndrome criteria was found in patients with SHZ.
Kornetova [32]	Inpatients aged 18 to 55 years living in Western Siberia.	1.38 (IQR 0.81–2.03)	SHZ patients showed lower TSH levels than controls.
Li [36]	Inpatients aged 18 to 60 years.	2.54 (IQR 1.67–4.20)	Increased fT3 and decreased serum TSH levels were independent risk factors for agitation in hospitalized patients with SHZ.
Li [44]	Adult inpatients diagnosed with SHZ	1.85 ± 0.93	Electroconvulsive therapy impaired hypothalamus–pituitary–thyroid axis: y THRT may help prevent amnesia.
Liang [26]	Chinese post-menopausal SHZ women patients	3.2 ± 2.9	TSH levels were superimposable on the prevalence of abnormal bone mineral density in SHZ women.
Lin [30]	SHZ outpatients and inpatients	1.5 ± 0.8	No differences in TSH levels were found among SHZ and healthy controls.
Makarow-Gronert [37]	Caucasian patients aged 12 to 18 years who were hospitalized in the Department of Adolescent Psychiatry.	2.12 ± 1.01	There may be a higher prevalence of thyroid dysfunctions in BD and MDD subgroups among adolescents than in SHZ.
Muzaffar [38]	Cannabis related SHZ in male patients aged 18 to 60.	1.61 ± 1.38	No differences in TSH, fT4, and fT3 serum levels were found among patients and healthy controls.
Ouyang [40]	Outpatients with first episode SHZ	1.69 ± 0.87	There was no significant difference in TSH, fT4, and fT3 levels between SHZ patients and healthy controls.
Petrikis [27]	Adult inpatients with SHZ	1.45 (IQR 0.26–3.49)	No differences in TSH levels among SHZ and healthy subjects were found; patients had lower levels of fT3 than controls.
Petruzzelli [33]	Adolescent inpatients admitted to Child and Adolescent Psychiatric Unit for first episode of SHZ.	2.0 ± 1.1	fT4 levels were significantly higher in SHZ patients than in those diagnosed with affective spectrum disorder.
Rao [18]	Adult SHZ inpatients	1.53 ± 1.11	Dopaminergic hyperactivity in SHZ may be related to a decrease in TSH hormone levels.
Roy [17]	SHZ outpatients who underwent TRH-TSH stimulation	2.6 ± 0.8	No differences were found between SHZ patients and healthy controls in TSH basal levels and TRH–TSH response.
Telo [28]	Adult patients with SHZ	2.15 ± 1.53	No differences were found between SHZ patients and healthy controls in TSH levels
Wahby [16]	Adult male patients who underwent TRH–TSH stimulation	2.92 ± 0.24	Schizodepressed patients appeared significantly different from MDD but closer to SHZ and healthy controls on the TRH test.
Wysokiński [23]	Hospitalized patients in acute phase evaluated at first entry	1.71 ± 1.49	In patients with schizophrenia, older patients had the lowest level of TSH.
Yazici [20]	Patients admitted to the psychiatric clinic for SHZ and followed up for 1 year.	1.35 ± 1.62	No differences in basal TSH levels were found among SHZ patients and healthy controls.
Zhai [41]	Patients with first episode of SHZ	1.72 ± 1.69	A higher central set point of thyroid homeostasis may be involved in the underlying mechanism of thyroid allostatic load in drug-naïve patients affected by first-episode of SHZ
Zhao [39]	Inpatients with a diagnosis of SHZ admitted with normal thyroid function tests	1.80 ± 1.52	Acute phase quetiapine treatment for schizophrenia patients was strongly associated with increased risk of developing new-onset hypothyroidism, with a clear dose–response association.
Zhao [42]	Inpatients with a diagnosis of SHZ admitted with normal thyroid function tests	1.83 ± 1.48	Impaired central set point may be involved in the mechanism by which quetiapine affects hypothalamus–pituitary–thyroid axis in acute phase of SHZ.
Zhu [34]	Chinese inpatients with SHZ	2.09 ± 1.48	Decreased fT3 and fT4 appear to be associated with SHZ symptoms.

TSH: thyrotropin-stimulating hormone; THRT: thyroid hormone replacement therapy; fT4: free thyroxine; fT3: free triiodothyronine; BD: bipolar disorder; MDD: major depressive disorder; TRH: thyrotropin-releasing hormone.

## Data Availability

No new data were created.

## Abbreviations

The following abbreviations are used in this manuscript:

FEDN	First-episode drug-naïve
SHZ	Schizophrenia
T3	Triiodothyronine
T4	Tetraiodothyronine
TH	Thyroid hormone
TRH	Thyrotropin-releasing hormone
TSH	Thyroid stimulating hormone
